# Evidence for cell turnover as the mechanism responsible for the transport of embryos towards the vagina in viviparous onychophorans (velvet worms)

**DOI:** 10.1186/s12983-019-0317-x

**Published:** 2019-06-07

**Authors:** Sandra Treffkorn, Oscar Yesid Hernández-Lagos, Georg Mayer

**Affiliations:** 1 Department of Zoology, Institute of Biology, University of Kassel, Heinrich-Plett-Str. 40, D-34132 Kassel, Germany; 2 Laboratorio de Biología Molecular, Escuela de Biología, Universidad Industrial de Santander, Carrera 27 #9, ciudad Universitaria, Bucaramanga, Santander Colombia

**Keywords:** Apoptosis, Caspase-3, Cell proliferation, Onychophora, Velvet worms, TUNEL

## Abstract

**Background:**

Onychophorans, commonly known as velvet worms, display a remarkable diversity of reproductive strategies including oviparity, and placentotrophic, lecithotrophic, matrotrophic or combined lecithotrophic/matrotrophic viviparity. In the placentotrophic species, the embryos of consecutive developmental stages are attached to the uterus via a placental stalk, suggesting they might be transported passively towards the vagina due to proximal growth and distal degeneration of tissue. However, this assumption has never been tested using specific markers. We therefore analyzed the patterns of cell proliferation and apoptosis in the genital tracts of two placentotrophic peripatids from Colombia and a non-placentotrophic peripatopsid from Australia.

**Results:**

All three species show a high number of apoptotic cells in the distal portion of the genital tract near the genital opening. In the two placentotrophic species, additional apoptotic cells appear in ring-like vestigial placentation zones of late embryonic chambers. While moderate cell proliferation occurs along the entire uterus in all three species, only the two placentotrophic species show a distinct proliferation zone near the ovary as well as in the ring-like implantation zone of the first embryonic chamber. In contrast to the two placentotrophic species, the non-placentotrophic species clearly does not show such regions of high proliferation in the uterus but exhibits proliferating and apoptotic cells in the ovarian stalks. While cell proliferation mainly occurs in stalks carrying maturating oocytes, apoptosis is restricted to stalks whose oocytes have been released into the ovarian lumen.

**Conclusions:**

Our results confirm the hypothesis that the uterus of placentotrophic onychophorans grows proximally but is resorbed distally. This is supported by the detection of a proximal proliferation zone and a distal degenerative zone in the two placentotrophic species. Hence, cell turnover might be responsible for the transport of their embryos towards the vagina, analogous to a conveyor belt. Surprisingly, the distal degenerative zone is also found in the non-placentotrophic species, in which cell turnover was unexpected. These findings suggest that the distal degenerative zone is an ancestral feature of Onychophora, whereas the proximal proliferation zone might have evolved in the last common ancestor of the placentotrophic Peripatidae.

**Electronic supplementary material:**

The online version of this article (10.1186/s12983-019-0317-x) contains supplementary material, which is available to authorized users.

## Background

**Fig. 1:**
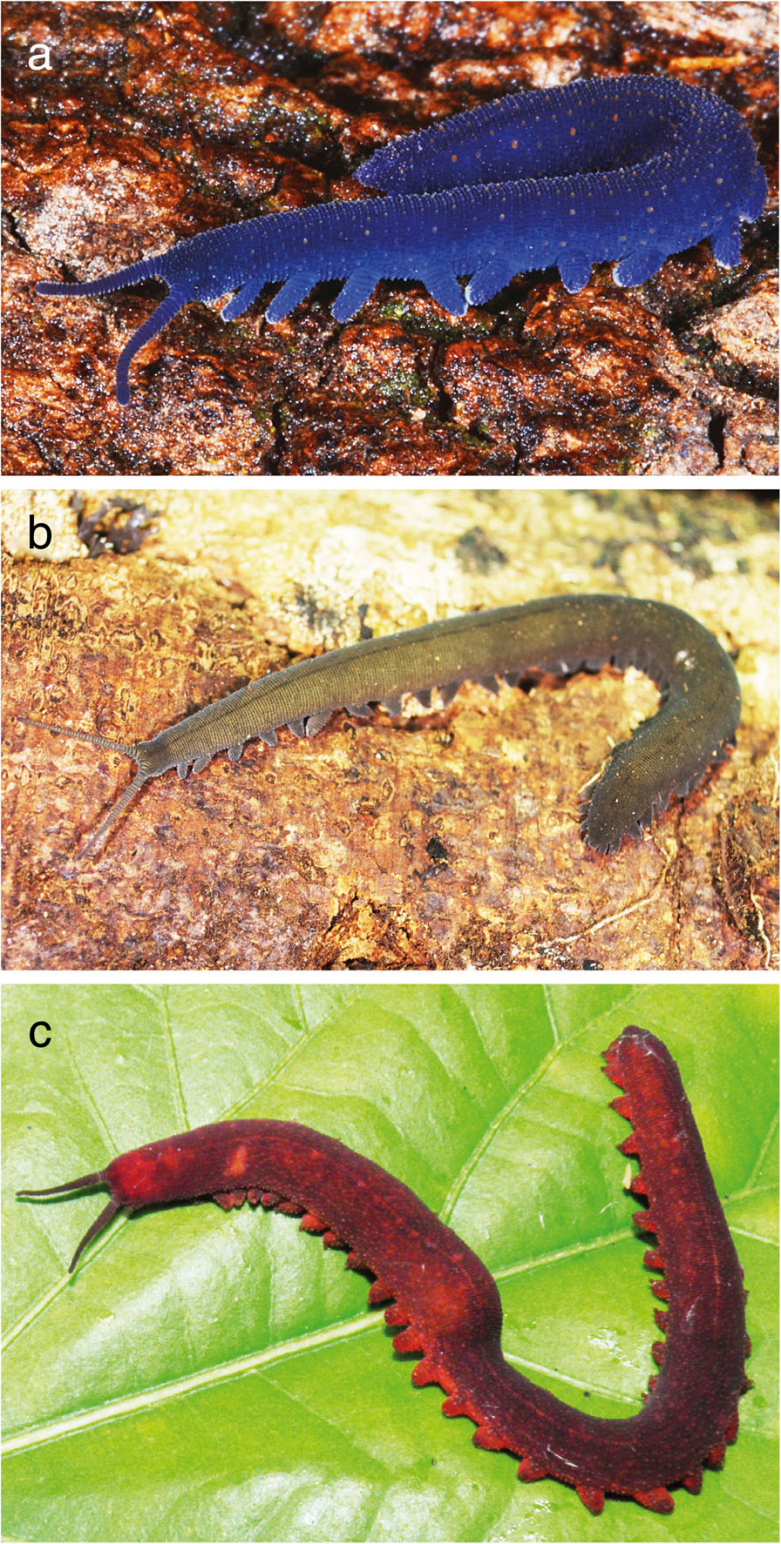
Photographs of specimens of *Euperipatoides rowelli* (**a**), gen. sp. 1 (**b**), and gen sp. 2 (**c**). Photograph of *E. rowelli* provided by Ivo de Sena Oliveira

**Fig. 2:**
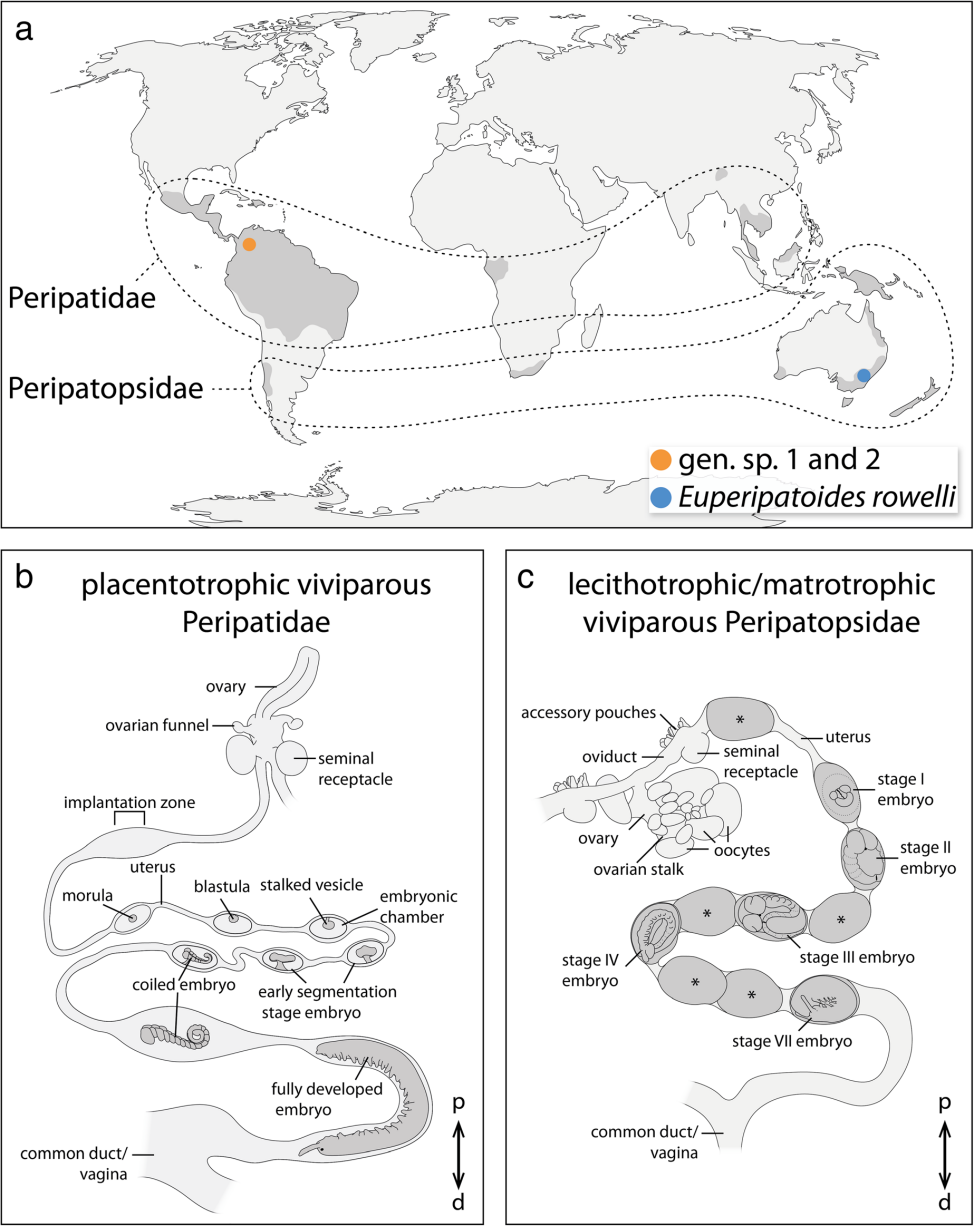
Localities of the species studied and structure of their genital tracts. **a** World map showing the distribution of Peripatidae and Peripatopsidae (areas in dark gray, modified from refs [2, 75]) and the localities of gen. sp. 1 and gen. sp. 2 collected in Colombia (orange dot), and *E. rowelli* collected in Australia (blue dot). **b, c** Diagrams of the reproductive tracts in placentotrophic viviparous peripatids (B, modified from refs [27, 39]) and combined lecithotrophic/matrotrophic viviparous peripatopsids (C). Only one horn of the paired uteri is shown in each diagram. Embryonic stages according to refs [15, 31], respectively. Asterisks indicate unfertilized eggs or undeveloped embryos, which occur frequently in *E. rowelli*. Abbreviations: d, distal; p, proximal

Onychophorans, also called “velvet worms” (Fig. 1a–c), are terrestrial invertebrates that can be found in tropical and temperate forests on landmasses that have resulted from the breakup of Gondwana (Fig. 2a) [1–4]. They are divided in two major groups, the Peripatidae with an equatorial distribution in West Africa, Central America and South-East Asia, and the Peripatopsidae with a distribution in South Africa, Australasia and Chile (Fig. 2a) [1, 2, 5]. Together with tardigrades (water bears) and arthropods (spiders, insects and allies), onychophorans comprise the Panarthropoda, which form the Ecdysozoa – the clade of molting animals – along with the Cycloneuralia (nematodes, priapulids and allies; [6, 7]; but see ref. [8] for a critical review of ecdysozoan phylogeny).

Even though the anatomy of onychophorans has changed little since the Early Cambrian [2], they have evolved a remarkable diversity of reproductive strategies (reviewed in ref. [2]). For example, oviparity occurs in some species of the Australian Peripatopsidae with yolky eggs that are surrounded by a vitelline envelope and a thick, sculptured chorion [2, 9–12]. On the other hand, placentotrophic viviparity is found in the neotropical Peripatidae (Neopatida *sensu *ref. [13]), which is characterized by a placental relationship between the yolkless embryo and the maternal uterine wall via a hollow stalk, which is a derivative of the dorsal extra-embryonic tissue (Fig. 2b) [2, 3, 14–23]. However, nourishment from the mother does not seem to be transferred via the stalk but rather via the embryonic surface [2, 18]. The remaining onychophoran species display various types of “ovoviviparity”, including lecithotrophic viviparity, matrotrophic viviparity and combined matrotrophic/lecithotrophic viviparity [2, 12, 22, 24–30]. While lecithotrophic onychophorans produce yolky eggs without any trophic interaction between the embryo and the mother [2, 12, 31–33], matrotrophic viviparous species have yolkless embryos that receive nourishments exclusively from the mother [24, 27–30, 34–38], and combined lecithotrophic/matrotrophic viviparous species produce yolky eggs that receive additional nourishments from the mother (Fig. 2c) [25, 31]. By mapping the types of nourishment supply on the onychophoran phylogeny [1], it has been suggested that the last common ancestor of Onychophora showed either lecithotrophic or combined lecithotrophic/matrotrophic viviparity [2]. Consequently, placentotrophic viviparity might be a derived feature of the Neopatida (neotropical Peripatidae) alone or the Neopatida plus the peripatid *Mesoperipatus tholloni* from tropical Africa, as the reproductive mode and embryonic development are unknown from the latter [1, 2].

Among these types of nourishment supply, placentotrophic viviparity is unique, as a similar developmental mode has not been described from any other invertebrate taxon so far [14, 20]. This placentation mode has been described to superficially resemble that of mammals, as the embryos develop a stalked placenta and the uterine wall surrounding the region of placental attachment becomes highly modified [14, 15, 20]. In contrast to mammals, however, the placental attachment persists only during the early growth and segment formation, while the more advanced developmental stages lie freely within the uterus [14, 15, 20, 39]. Moreover, the paired uteri of placentotrophic viviparous females each contain a series of embryos of increasing age with the most immature embryo being located near the ovary and the largest, almost fully developed fetus near the genital opening [14, 20]. This situation leads to the paradox that the embryos – although attached to the uterine wall in early developmental stages – must move towards the genital opening during the course of their development [14, 20]. It has been hypothesized previously [14, 20] that the proximal end of the uterus is a constantly growing region, which receives new implants at regular intervals, while excess tissue is resorbed near the genital opening. It has been proposed that this combination of growth and resorption of tissue is unique to placentotrophic species of onychophorans [20], since embryos of the remaining species are not attached to the maternal uterus and thus move freely within its lumen via contractions of the uterus musculature [2, 20]. Similar patterns of growth and resorption of uterine tissue have never been reported from non-placentotrophic onychophorans [14, 20]. Hence, it has been proposed that the complementary growth and resorption of uterine tissue is a derived feature of the placentotrophic onychophorans [20].

However, this hypothesis has never been tested by using specific markers for cell proliferation and controlled cell death [40–43]. In this study, we therefore investigated patterns of cell proliferation and apoptosis – a specific type of controlled cell death [41–43] – in female genital tracts of two undescribed species of the placentotrophic Peripatidae from Colombia and the lecithotrophic/matrotrophic peripatopsid *Euperipatoides rowelli* from Australia. To detect cell proliferation, we used an antibody directed against phospho-histone H3 as a mitosis marker. Apoptotic cells were detected using the terminal deoxinucleotidyl transferase-mediated dUTP nick end labeling (TUNEL) technique, which labels free 3′-OH ends of the degrading DNA of apoptotic cells [44]. Additionally, we used an antibody directed against the activated form of caspase-3, a highly conserved protein that is essential for apoptosis in metazoans [45]. The combination of these markers has been used previously to detect patterns of apoptosis and cell proliferation in embryos of *E. rowelli* and the placentotrophic viviparous peripatid *Principapillatus hitoyensis* [39].

The results of this study might help to clarify whether the complementary growth and resorption of uterine tissue is an ancestral feature of all onychophorans or a derived feature of the placentotrophic Peripatidae, which has evolved along with the placental attachment of embryos to the uterine wall. Hence, the comparison of cell proliferation and apoptosis patterns in the genital tracts of *E. rowelli* and the placentotrophic Peripatidae might provide insights into the evolution of reproductive strategies in onychophorans.

## Results

### Comparative anatomy of the genital tracts

The female reproductive tract of all investigated species consists of an ovary, short paired oviducts associated with seminal receptacles, paired tubular uteri, and a common duct or vagina (Fig. 2b, c). Throughout the following descriptions, the terms “proximal” and “distal” refer to parts of the genital tract near the ovary or genital opening, respectively (*sensu* ref. [20]; but note that these terms are used in opposite orientation in other animals such as the fruit fly *Drosophila melanogaster* [46]). Each horn of the paired tube-like uteri typically contains a series of embryos of increasing age, the youngest one being located proximally near the ovary and the oldest one distally near the genital opening (Fig. 2b, c). In *E. rowelli*, this succession might occasionally be interrupted due to the occurrence of embryos that have not developed beyond stage I (Fig. 2c; staging according to ref. [31]).

The paired tubular ovaries of the placentotrophic Peripatidae have a smooth external surface without externally visible oocytes (endogenous ovarian type *sensu* ref. [47]; Fig. 2b). The ovaries lead into short paired oviducts, which are associated with a pair of ovarian funnels and seminal receptacles (*sensu* ref. [48]; Fig. 2b). The presence of embryos within each uterus horn is marked externally by visible swellings of the uterine walls, which comprise the embryonic chambers and retain their shape even after the embryos have been dissected from the individual chambers. The yolkless embryos of the placentotrophic Peripatidae are not surrounded by any secreted envelopes, but instead become attached to the maternal uterus wall via a placental stalk, which persists until segmentation is completed (Fig. 2b). The uterus of these species is subdivided into an upper region, containing a succession of embryos from early cleavage stage to coiled stage embryos (staging according to refs [15, 17, 18]), which are attached to the uterus via a placental stalk, and a lower region containing large embryos that have lost their connection to the uterus and lie freely within its lumen (Fig. 2b).

In contrast to this, the grape-like ovary (exogenous ovarian type *sensu* ref. [47]) of *E. rowelli* bears externally visible oocytes of different sizes, the largest of which are raised above the ovarian surface via stalks (Fig. 2c). Additionally, the seminal receptacles of *E. rowelli* are associated with accessory pouches while ovarian funnels (*sensu* ref. [49]) are absent. In contrast to the placentotrophic Peripatidae, the embryos of *E. rowelli* are surrounded by a thin vitelline envelope and a chorion that persist until birth, and they lie freely in the lumen of the uterus (Fig. 2c). Even though the presence of embryos within the uterus is also marked by externally visible swellings, in contrast to the embryonic chambers of the placentotrophic Peripatidae, these swellings do not have a defined shape, which would persist after dissecting the embryos. Instead, the uterine tissue is stretched as the embryos pass through the lumen and regains its original shape after the embryos are transported further towards the genital opening.

### Cell proliferation patterns in the reproductive tracts

**Fig. 3:**
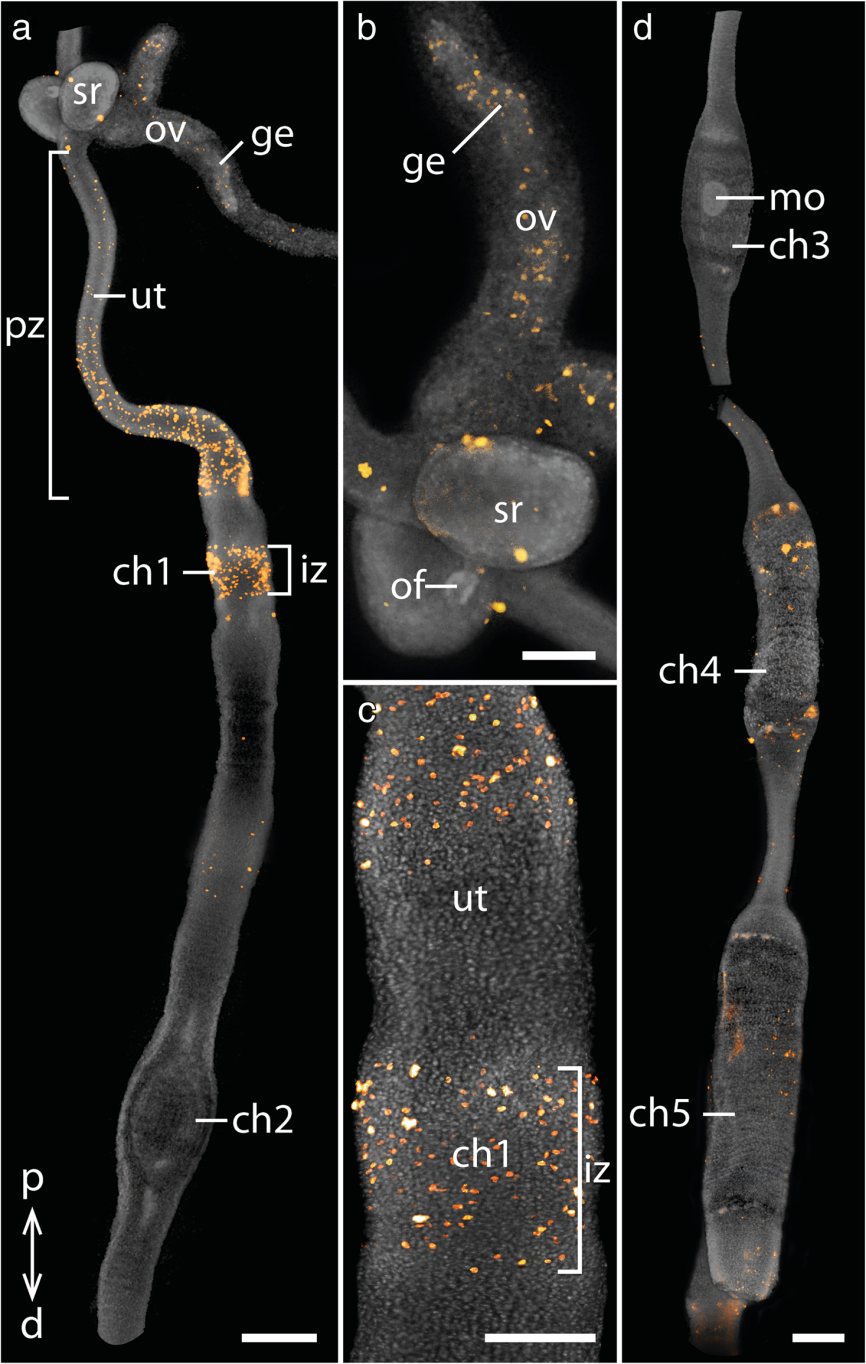
Cell proliferation in the genital tract of the placentotrophic peripatid gen. sp. 2. Nuclei stained with DAPI are represented in gray, condensing DNA and chromosomes of dividing cells (α-PH3 labeling) in orange. Proximal is up in all images. **a** Proximal part of the genital tract including the ovary and the first two uterine chambers containing early embryos. **b** Detail of the ovary with seminal receptacles and ovarian funnels. Note that the signal in the ovaries is restricted to the germinal epithelium. **c** Detail of the first chamber with an implantation zone. **d** Third to fifth chambers containing a morula and early segmentation stage embryos. Abbreviations: ch1–ch5, uterine chambers 1 to 5 with embryos of subsequent developmental stages; d, distal; ge, germinal epithelium; iz, implantation zone; mo, morula; of, ovarian funnel; ov, ovary; p, proximal; pz, proximal proliferation zone; sr, seminal receptacle; ut, uterus. Scale bars: A, D: 500 μm; B, C: 200 μm

**Fig. 4:**
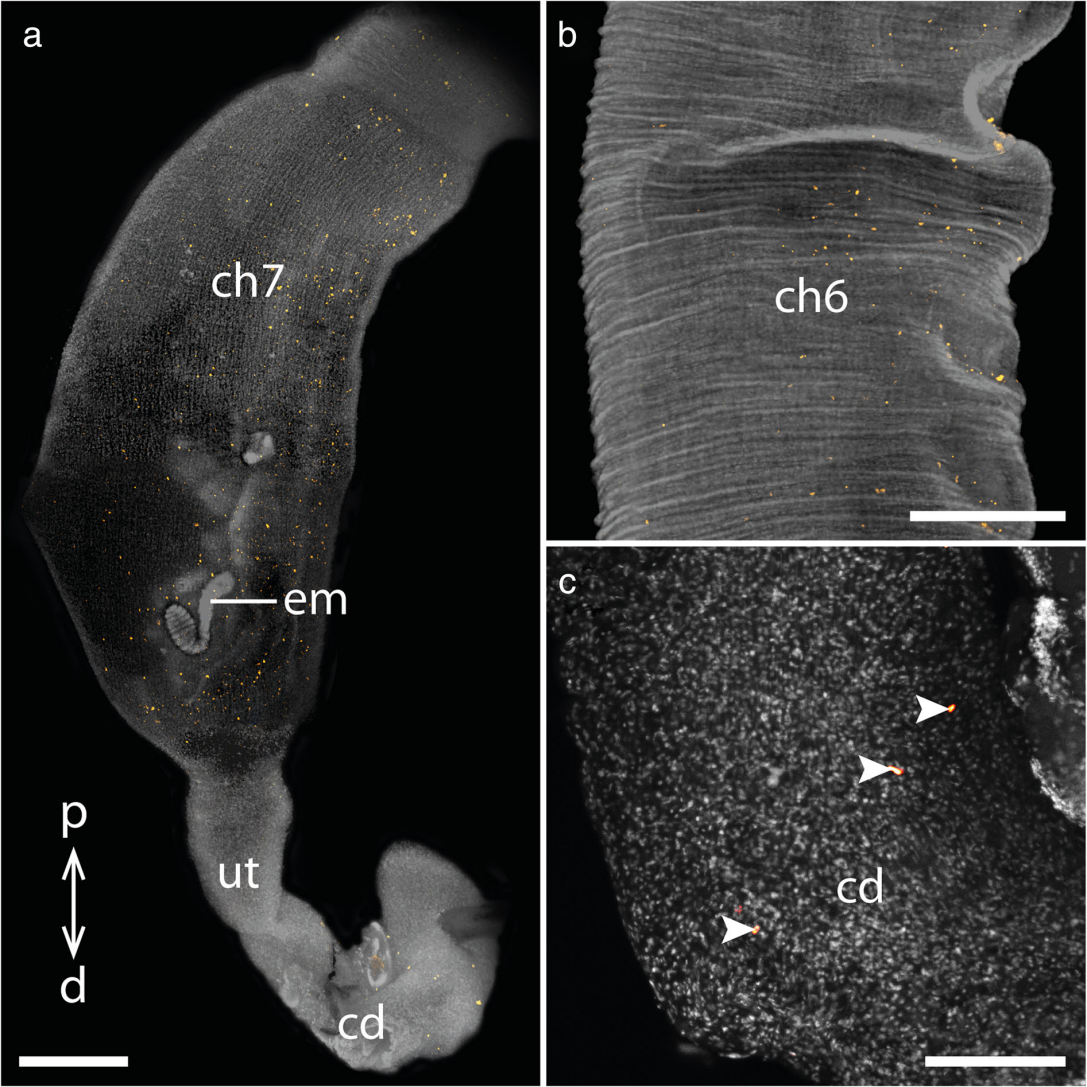
Cell proliferation in the genital tract of the placentotrophic peripatid gen. sp. 2. Nuclei stained with DAPI are represented in gray, condensing DNA and chromosomes of dividing cells (α-PH3 labeling) in orange. Proximal is up in all images. **a** Distal part of the genital tract including the uterine chamber containing a fully developed embryo and the common duct/vagina. **b** Detail of the sixth chamber containing an almost fully developed embryo. **c** Detail of the common duct/vagina. Abbreviations: cd, common duct/vagina; ch6 and ch7, uterine chambers containing embryos of subsequent developmental stages; d, distal; em, fully developed embryo; p, proximal; ut, uterus. Scale bars: A: 1 mm; B: 500 μm; C: 200 μm

We analyzed cell proliferation patterns in the genital tracts of several adult females of two species of the placentotrophic Peripatidae from Colombia and the lecithotrophic/matrotrophic viviparous species *E. rowelli* (Table 1). Immunolabeling with an antibody against the mitosis marker phospho-histone H3 in the reproductive tracts of the placentotrophic Peripatidae revealed similar patterns in both investigated species (Figs. 3a-d, 4a-c; Additional file 1). Proliferating cells are distributed along the entire genital tracts, including the germinal epithelium of the ovaries and the seminal receptacles (but note that, due to the method used, we cannot rule out the possibility that the signal detected in the ovaries might be the result of both mitosis and meiosis). A particularly strong signal appears in the narrow proximal part of the uterus near the ovaries, with an increasing intensity towards the first embryonic chamber containing the youngest embryo (Fig. 3a–c). Additionally, a strong ring-shaped signal is detected in the center of this first uterine chamber (Fig. 3a, c). In the following part of the uterus, including the second and third embryonic chambers, only a small number of dividing cells is detected, until an increased signal appears again in the wall of the fourth chamber containing an early segmenting embryo that has started to elongate (Fig. 3d). This signal persists throughout the remaining upper and the entire lower region of the uterus including the chamber containing the largest, almost fully developed embryo (Figs. 3d, 4a, b). In the distal-most part of the uterus as well as the common duct or vagina, only a small number of dividing cells is detected (Fig. 4a, c).

**Table 1:** List of onychophoran species studied with corresponding locality data and number of genital tracts used for individual experiments

	Species	Locality	Number of females used for	
			TUNEL/anti-PH3	anti-caspase-3
Peripatidae	^a^gen. sp. 1	6°30′08.8″N, 73°06′08.5″W, 1710 m, Santander Department, Los Santos, Colombia	2	1
	^a^gen. sp. 2	5°48′55.4″N, 73°57′33.2″W, 1819 m, Santander Department, Florián, Colombia	2	1
Peripatopsidae	*Euperipatoides rowelli*	35°26′S, 149°33′E, 954 m, Tallaganda State Forest, New South Wales, Australia	6	2

^a^ Undescribed species

**Fig. 5:**
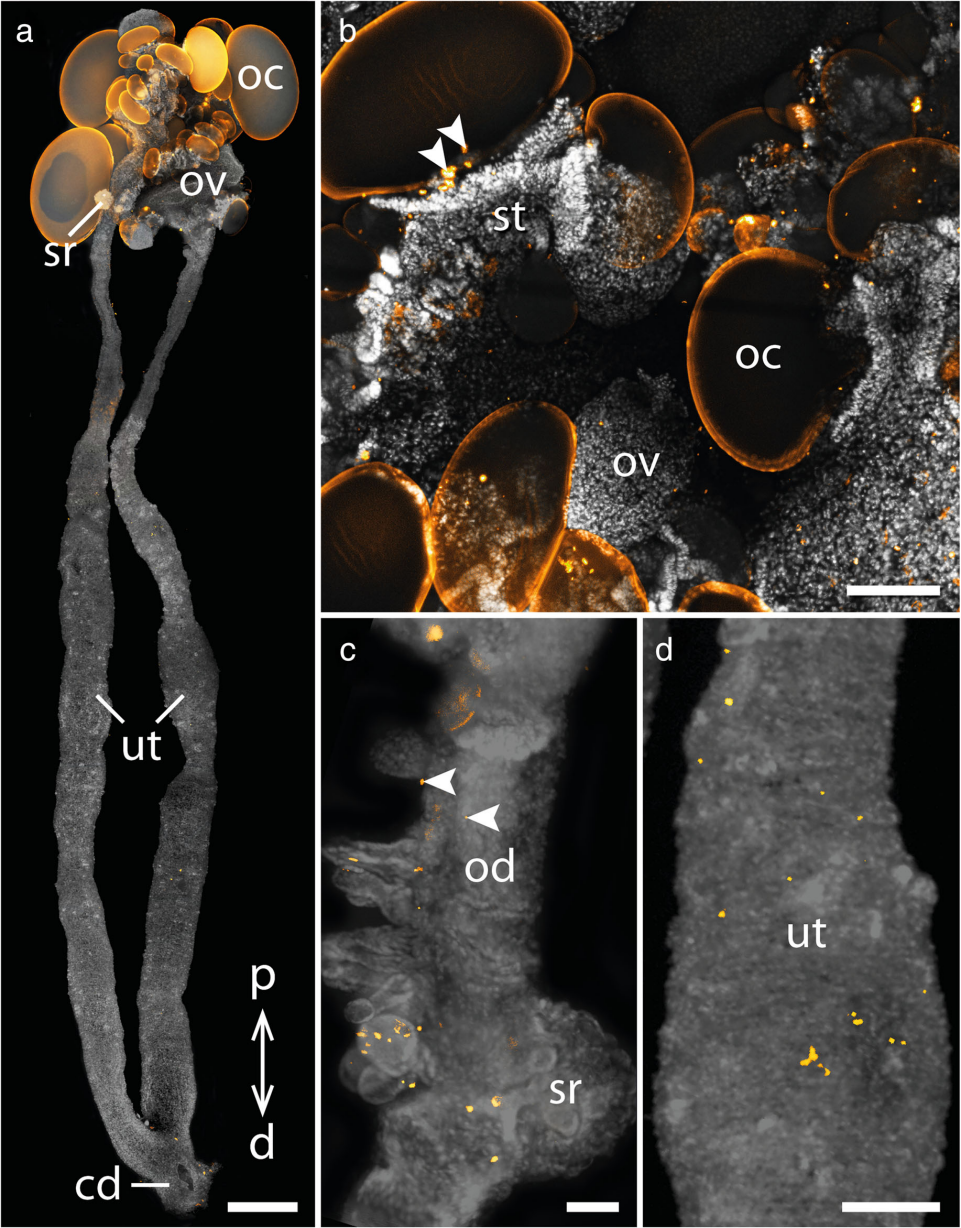
Cell proliferation in the genital tract of *E. rowelli*. Nuclei stained with DAPI are represented in gray, condensing DNA and chromosomes of dividing cells (α-PH3 labeling) in orange. Proximal is up in all images. **a** Overview of a complete genital tract. **b** Detail of the ovary containing oocytes of different sizes. Arrowheads point to proliferating cells in the stalk of an intermediate-sized oocyte. **c** Oviduct with seminal receptacle. Note that only a small number of dividing cells is detectable in the oviduct (arrowheads). **d** Proximal part of the uterus containing the youngest embryo. Abbreviations: cd; common duct/vagina; d, distal; oc, oocyte; ov, ovary; p, proximal; sr, seminal receptacle, ut, uterus. Scale bars: A: 1 mm; B, D: 200 μm; C: 100 μm

Similar to the placentotrophic Peripatidae, proliferating cells appear throughout the entire genital tract of *E. rowelli*, including the ovary and the paired oviducts and the uteri (Fig. 5a–d). Some signal occurs in the ovarian stalks, which in this species carry maturating oocytes and raise them above the ovarian surface. Additional signal is seen in the primary egg membrane (*sensu* ref. [47]) of large oocytes, which might be due to autofluorescence, as it is also seen in the controls without the antibody. The short oviducts leading from the ovary to the seminal receptacles only show a small number of proliferating cells (Fig. 5c), whereas an increased signal is detected in the paired uteri near the seminal receptacles and distal of those (Fig. 5a, c). In contrast to the placentotrophic Peripatidae, dividing cells are distributed evenly throughout the proximo-distal extent of the uteri without an increased signal at the proximal end near the ovary (Fig. 5a, c, d).

### Apoptosis patterns in the reproductive tracts

**Fig. 6:**
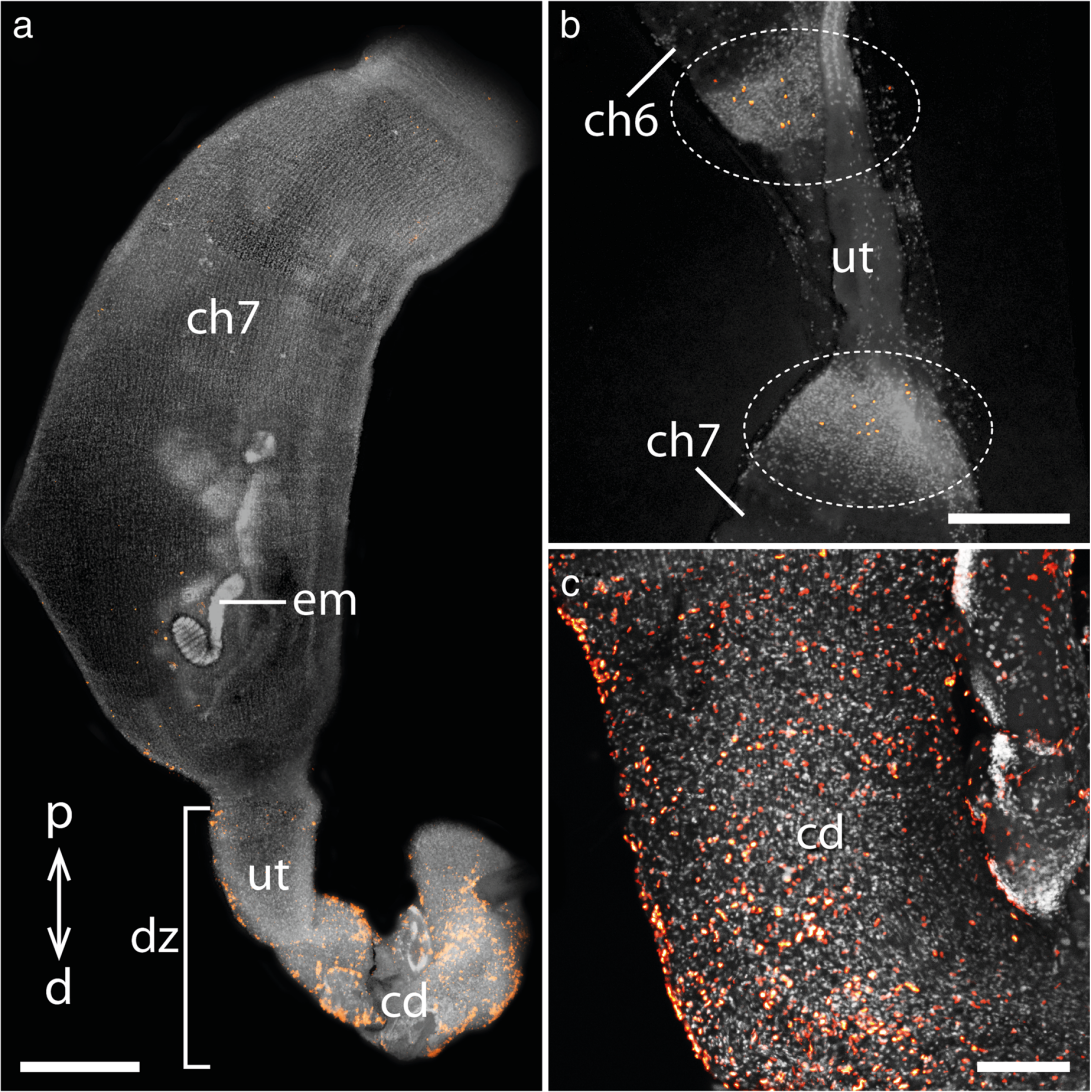
Apoptosis in the genital tract of the placentotrophic peripatid gen. sp. 2. Nuclei of TUNEL-positive cells are marked in orange, nuclei stained with DAPI are represented in gray. Proximal is up in all images. **a** Distal part of the genital tract, including the chamber with an almost fully developed embryo, as well as the common duct or vagina. **b** Detail of the uterus portion connecting the sixth and seventh chambers. Note the apoptotic cells in repeated regions of condensed tissue (dotted ovals). **c** Detail of the common duct/vagina. Abbreviations: cd, common duct/vagina; ch6 and ch7, uterine chambers containing embryos of subsequent developmental stages; d, distal; dz, distal degeneration zone; em, fully developed embryo; p, proximal; ut, uterus. Scale bars: A: 1 mm; B, C: 200 μm

In addition to cell proliferation, we detected apoptosis using TUNEL and anti-caspase-3 labeling (Table 1). In contrast to cell proliferation, apoptosis is absent from the proximal part of the uterus in the placentotrophic Peripatidae, including the ovaries and the upper region of paired uteri containing the embryos that are attached to the uterus via placental stalks (Additional file 2). In the lower portion of the paired uteri containing the two oldest embryos, a small number of apoptotic cells is detected scattered throughout the uterine walls (Fig. 6a). Additionally, apoptotic cells are detected in distinct rings of condensed tissue, which appear at regular intervals throughout the lower region of the uterus containing the two largest embryos (Fig. 6b). A high number of apoptotic cells appears in the short portion of the uteri distal to the uterine chamber containing the largest embryo as well as in the distal common duct belonging to the vagina (Fig. 6a, c).

**Fig. 7:**
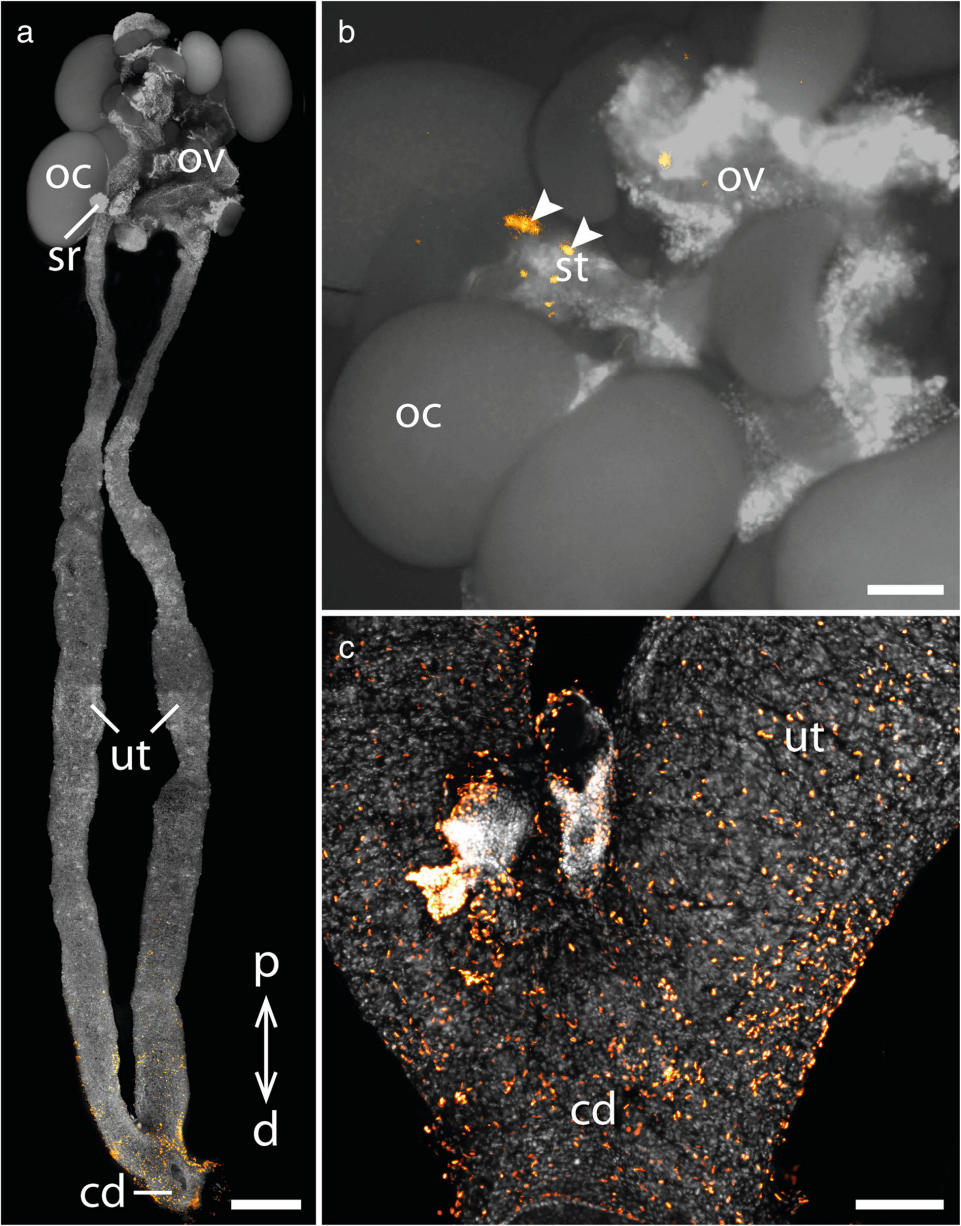
Apoptosis in the genital tract of *E. rowelli*. Nuclei of TUNEL-positive cells are marked in orange, nuclei stained with DAPI are represented in gray. Proximal is up in all images. **a** Overview of a complete genital tract. **b** Detail of the ovary containing oocytes of different sizes. Arrowheads point to apoptotic cells in an empty stalk. **c** Detail of the common duct/vagina. Abbreviations: cd, common duct/vagina; d, distal; oc, oocyte; ov, ovary; p, proximal; sr, seminal receptacle; ut, uterus. Scale bars: A: 1 mm; B, C: 200 μm

Similarly, apoptosis is absent from the proximal two thirds of the genital tract of *E. rowelli*, except for individual apoptotic cells appearing in the empty stalks of the ovaries (Fig. 7a, b; Additional files 2, 3A). From the distal third of the paired uteri onwards an increasing number of apoptotic cells is detected with the highest number being located in the fused common duct leading to the genital opening (Fig. 7a, c; Additional files 2, 3B).

## Discussion

### Conveyor belt-like transport of embryos in the uterus of placentotrophic species

**Fig. 8:**
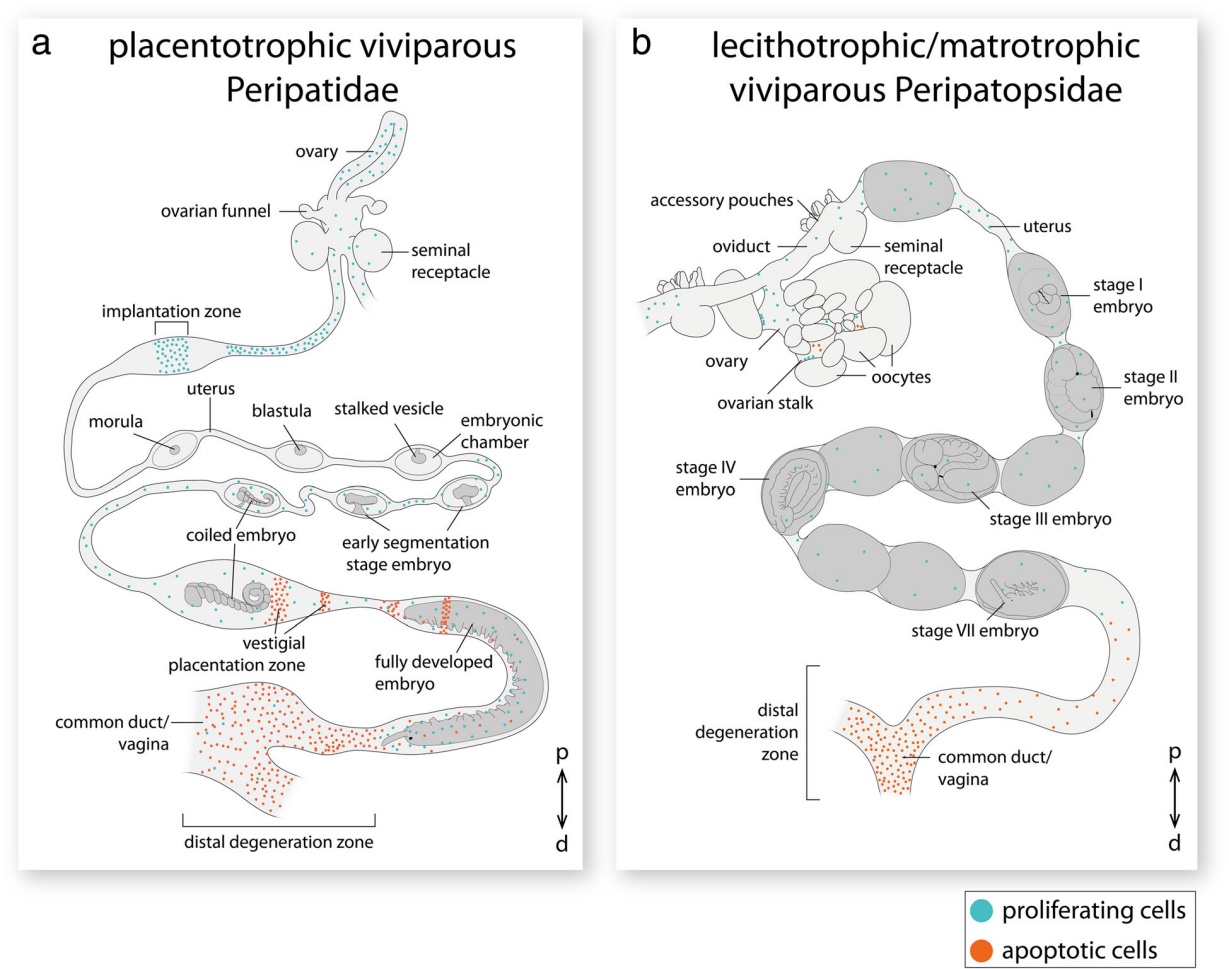
Comparison of apoptosis (orange) and cell proliferation patterns (cyan) in the genital tracts of placentotrophic (**a**) and combined lecithotrophic/matrotrophic (**b**) velvet worms. Abbreviations: d, distal; p, proximal

The detection of cell proliferation in the two species of placentotrophic Peripatidae from Colombia revealed that the short proximal part of the uterus contains a distinct proliferation zone as indicated by the occurrence of an increased number of proliferating cells compared to the surrounding tissue (Fig. 8a). In the middle of the first uterine chamber, an additional area of high cell proliferation appears, which corresponds in position to the implantation zone of the early embryo. The implantation of the embryo is typically accompanied by a modification of the uterine wall and the development of an epithelial sac around the early embryo [15, 20]. These processes might be characterized by an increased cell division in the area of the implantation zone. In contrast to cell proliferation, apoptosis is completely missing in the proximal part of the uterus containing embryos that are still attached to the uterus via a stalk (Fig. 8a). The majority of apoptotic cells are restricted to the short distal exit duct leading to the genital opening, indicating that this area is rapidly degrading. The presence of a distinct proximal proliferation zone together with a distal degenerative zone suggests that on the one hand the uterus is constantly growing at the proximal end, while on the other hand tissue is resorbed at a similar rate at the distal end (Fig. 8a).

This confirms previous, histological and light microscopical observations on the genital tracts of the placentotrophic onychophorans *Macroperipatus torquatus* and *Epiperipatus trinidadensis* [14, 20], where the proximal uterus might comprise a constantly growing region with new fertilized eggs becoming implanted at regular intervals, while resorption of the uterus wall is evident throughout the lower uterus containing the two largest embryos [14, 20]. In contrast to this, no evidence for growth or resorption of uterine tissue was found in *Epiperipatus acacioi* [15]. However, Campiglia and Walker [15] stated that the length of the uterus of pregnant females varies according to the number of offspring a female has given birth to during her life. Furthermore, they observed regions of the uterus near the vagina, in which the lining epithelium seems to disintegrate [15], indicating that growth and resorption of uterine tissue might take place in *E. acacioi* as well.

While the overall pattern of proximal growth in conjunction with distal resorption of the uterine wall is similar in the two placentotrophic peripatids from Colombia and the previously studied representatives of placentotrophic Peripatidae [14, 20], our analysis of patterns of cell proliferation and apoptosis also revealed some differences to the previous observations. For example, Anderson and Manton [20] state that they did not observe any growth of the uterus other than proximal to the first uterine chamber in *M. torquatus* and *E. trinidadensis*. While our results confirm that cell proliferation is almost completely absent in the uterine portion associated with embryos up to the stalked vesicle stage (Fig. 8a), cell proliferation seems to be initiated again in the walls of chambers containing large, elongating embryos and persists in the distal-most chamber containing the largest, almost fully developed embryo. Furthermore, we found that apoptotic cells appear at regular intervals in more or less repeated, narrow rings of condensed tissue throughout the distal portion of the uterus containing the two largest embryos (Fig. 8a). These areas correspond in position to the vestigial placentation zones from prior births that have been described in *Macroperipatus torquatus* and *Epiperipatus trinidadensis* [20], although similar regions were not detected in *E. acacioi* [15]. Moreover, Anderson and Manton [20] state that resorption of the uterine wall appears throughout the entire lower portion of the uterus, containing the two oldest embryos. In contrast to this, apart from apoptotic cells in the vestigial placentation zones, only a small number of apoptotic cells appears in the chambers containing the two oldest embryos in the placentotrophic Peripatidae from Colombia, indicating that the resorption of the uterus is restricted to the distal common duct in these species. Taken together, these differences indicate that there might be species-specific variations in the structure of uterine tissue and the cell turnover processes taking place, which corresponds with previously observed structural differences in the reproductive tracts of different representatives of the placentotrophic Peripatidae [14, 15, 20].

Irrespective of these variations, our results support the hypothesis that the uterus of the placentotrophic species is constantly growing at the proximal end (i.e., near the ovary), while the tissue is resorbed distally (i.e., near the genital opening) [14, 20], thus generating space for new implants and moving the attached embryos towards the vagina in a conveyor-like fashion. This process might thus be the result of constant cell turnover mediated by cell proliferation and apoptosis in restricted areas of the genital tract.

Interestingly, our results further revealed cell proliferation along the entire proximo-distal extent of the paired uteri in the lecithotrophic/matrotrophic peripatopsid *E. rowelli*, whereas a distinct proliferation zone is missing. This indicates that the uterus of *E. rowelli* is not growing in a defined, proximal region but rather along its entire length, similar to the distal half of the uteri of the placentotrophic species in which the embryos have lost their connection to the uterus and lie freely within its lumen (Fig. 8b) [14, 17, 20]. This is similar to the embryonic development of Onychophora, where proliferating cells are distributed in a random fashion along the entire length of the germ band rather than being restricted to a localized proliferation zone [50]. Surprisingly, however, the distal part of the uterus of *E. rowelli*, including the fused duct or vagina, showed a high number of apoptotic cells, similar to that of the placentotrophic Peripatidae. Thus, while there is no evidence for the existence of a proximal proliferation zone in *E. rowelli*, the occurrence of a large number of apoptotic cells in all specimens studied (*n* = 6) indicates the presence of a distinct distal degeneration zone.

In summary, our data revealed that cell proliferation occurs along the entire genital tract of all species investigated whereas apoptosis is restricted to a distal degeneration zone. In contrast to this, a distinct proximal proliferation zone is only present in the placentotrophic Peripatidae. Thus, our results indicate that the distal degenerative zone of the uterus is an ancestral feature of Onychophora, while the proximal proliferation zone might have evolved in the last common ancestor of the placentotrophic Peripatidae [20]. Similar proliferation zones are a common feature during embryonic development of many animals [21, 51–55], but also in adult tissues with continuous cell turnover. For example, numerous proliferation zones have been described to occur in the brain and central nervous system of many adult vertebrates and invertebrates [56–59]. Furthermore, distinct proliferation zones appear in the midgut of adult tardigrades [60] as well as in the intestinal epithelium of adult mammals [61, 62]. Interestingly, embryos of the placentotrophic Peripatidae become implanted only distal of the proliferation zone, indicating that the tissue of the proliferation zone might consist of undifferentiated cells which might act as a source of uterine tissue progenitors. However, in order to clarify whether always the same stem-like cells divide, further studies are necessary to visualize cell proliferation in vivo, for example by using the incorporation of DNA replication markers such as EdU (5-ethynyl-2′-deoxyuridine) or BrdU (5-bromo-2′-deoxyuridine) in conjunction with antibody labeling [50, 60, 63–65].

### Apoptosis is responsible for degeneration of ovarian stalks in the exogenous ovary

In addition to the distinct patterns in the uterus, the cell proliferation marker used yielded signal in the ovaries of all investigated species. This signal might originate from oogonia situated within the germinal epithelium, which undergo mitosis and eventually give rise to cells that begin meiosis and become early maturing oocytes [47]. Since the antibody directed against phospho-histone H3 labels both mitotic and meiotic cells, the signal detected in the ovaries might be the result of a mixture of mitotic and meiotic activity.

Additionally, we identified proliferating and apoptotic cells in the ovarian stalks of *E. rowelli* (Fig. 8b). While proliferating cells were detected only in the stalks of medium-sized oocytes, apoptotic cells appear exclusively in empty stalks, the oocytes of which have been detached and have passed into the ovarian lumen [31, 66]. These results indicate that the stalks arise and grow out due to increased cell proliferation as the oocytes mature and increase in size, thus raising the maturating oocytes above the ovarian surface. This is reminiscent of what has been described from other onychophorans bearing the exogenous ovarian type, i.e., with oocytes projecting into the hemocoel on stalks formed by surrounding germinal tissue [12, 33, 47, 67]. Our data further show that, as soon as the mature oocytes have lost their connection to the stalks and have moved into the lumen of the genital tract, the stalks disintegrate as evidenced by the occurrence of apoptotic cells in the empty stalks. This suggests that the formation and degeneration of stalks is regulated by a combination of cell proliferation and apoptosis.

In contrast to this, apoptosis is completely absent from the endogenous ovary (i.e., with oocytes residing inside the ovary rather than protruding into the hemocoel [47]) of the two placentotrophic Peripatidae from Colombia, which corresponds to the complete reduction of stalks in this ovarian type and relocation of maturating oocytes to the germinal epithelium lining the ovarian lumen [47]. The detected anti phospho-histone H3 signal in the germinal epithelium is most-likely due to mitotic and meiotic activity of oogonia in this cell layer. Thus, a pattern of cell proliferation and apoptosis, which is associated with growth and degeneration of stalks in *E. rowelli*, does not occur in the placentotrophic Peripatidae from Colombia.

Based on an outgroup comparison with arthropods, some of which display exogenous ovaries [68–71], it has been proposed that the endogenous ovarian type might have evolved from the ancestral exogenous type [47]. This transformation was most likely accompanied by a complete loss of the ability to produce stalks, which is supported by our data. In this respect, it would be interesting to study the corresponding patterns of cell proliferation and apoptosis in the onychophoran species bearing the pseudoendogenous ovarian type, in which the stalks are still present inside the ovary but do not grow out into the hemocoel [24, 47]. We would expect apoptotic cells to occur in the stalk as soon as the mature oocyte has been released into the ovarian lumen, whereas proliferating cells might be less abundant due to the decreased size of stalks [47].

## Conclusions

Our results confirm a previous assumption that the genital tracts of the placentotrophic viviparous Peripatidae is constantly growing at the proximal end while tissue is resorbed at the distal end near the genital opening, thus retaining a constant length of the uterus while the embryos are transported towards the genital opening in a conveyor-like fashion. Surprisingly, we also detected a similar distal degenerative zone (albeit no proximal proliferation zone) in the genital tract of the lecithotrophic/matrotrophic viviparous species *E. rowelli*, in which the embryos are not attached to the uterine wall and thus cell turnover was unexpected. These findings suggest that the distal degenerative zone might be an ancestral feature of onychophorans, whereas the proximal proliferation zone might be a derived feature of the placentotrophic Peripatidae. To test this hypothesis, future studies should focus on the existence of the distal degenerative zone in additional representatives of Peripatopsidae and South-East Asian Peripatidae as well as the proximal proliferation zone in the peripatid *Mesoperipatus tholloni* from tropical Africa. The occurrence of a distinct pattern of cell proliferation and apoptosis in the stalks of the exogenous ovary of *E. rowelli* indicates that these processes are involved in the formation and degeneration of stalks. The absence of a corresponding pattern in the endogenous ovary of the placentotrophic Peripatidae confirms the proposed [47] loss of stalks in this onychophoran lineage. To better understand the evolution of embryonic nutrition and ovarian types across the Onychophora, it would be necessary to investigate the corresponding patterns of apoptosis and cell proliferation in species with other modes of nourishment supply including oviparity, lecithotrophic viviparity, and matrotrophic viviparity.

## Methods

### Specimens and sample preparation

Females of the combined lecithotrophic/matrotrophic viviparous species *Euperipatoides rowelli* Reid, 1996 (Peripatopsidae) from Australia and two undescribed placentotrophic viviparous species of Peripatidae (Neopatida) from Colombia, gen. sp. 1 and gen. sp. 2, were studied (Fig. 1a–c; Table 1). Females of *E. rowelli* (Fig. 1a) are usually between 30 and 60 mm in length. Body size of gen sp. 1 (Fig. 1b) ranges from 20 to 22 mm in virgin/juvenile females up to 40–85 mm in mature/adult females, while females of gen. sp. 2 (Fig. 1c) typically reach body sizes between 25 and 32 mm (virgin/juvenile females) and 90–110 mm (mature/adult females). The animals were collected and exported under the following permits: (1) Corporación Autónoma Regional para la Defensa de la Meseta de Bucaramanga (CDMB, permit number: PC-0002-2014), (2) Autoridad Nacional de Licencias Ambientales (ANLA), and (3) National Parks & Wildlife Service New South Wales (permit number: SL101720). Specimens of gen. sp. 1 and gen. sp. 2 were collected in a shadowed coffee plantation under rotten logs and leaf litter, and maintained in plastic boxes at 17–25 °C following the instructions described in refs [72, 73]. Specimens of *E. rowelli* were collected from decaying logs in October 2016 and maintained as described previously [72–74]. Adult females were anaesthetized with chloroform vapor for 20 s. The genital tracts were dissected and fixed overnight in 4% paraformaldehyde in phosphate-buffered saline (PBS 0.1 mol/L, pH 7.4) at room temperature. After several washes in 1 mL PBS (5 × 5 min; room temperature), some uteri were preserved in 1 mL PBS with sodium azide (0.05%) at 4 °C, while the others were dehydrated in an increasing methanol series (7 min each in 25, 50, 75%, 2 × 100% MeOH at room temperature) and kept in 1 mL absolute methanol at − 20 °C. Embryos of *E. rowelli* were staged according to ref. [31], those of gen. sp. 1 and gen. sp. 2 according to refs [15, 17, 18].

### Detection of fragmented DNA or cell death

The genital tracts stored in absolute methanol were rehydrated stepwise in PBS (7 min each in 75, 50, 25% MeOH, 2 × PBS at room temperature), those stored in PBS with sodium azide were rinsed several times in 1 mL PBS (5 × 5 min). Afterwards, they were incubated in 1 mL of 0.1 M sodium citrate (pH 6.0) for 30 min at 70 °C and rinsed in 1 mL PBS containing 0.3% Triton X-100 (PBS-Tx) at room temperature. Apoptotic cells were detected using the In Situ Cell Death Detection Kit (TMR red; Roche, Mannheim, Germany) according to the manufacturer’s protocol. The genital tracts were incubated in 450 μL labeling solution containing 50 μL enzyme solution for 2 h at 37 °C in a hybridization oven with gentle shaking. Positive controls were performed by treating the tissue with 0.25 mg/ml DNAse I (Roche) for 30 min at room temperature prior to the TUNEL labeling procedure. In these controls, all nuclei were TUNEL positive (Additional file 4).

### Immunohistochemistry and DNA labeling

After the application of the In Situ Cell Death Detection Kit, the genital tracts were washed several times in 1 mL PBS (2 × 5 min, 3 × 10 min; room temperature) and pre-incubated in 1 mL 5% normal goat serum (NGS; Sigma-Aldrich, St. Louis, MO, USA) in PBS-Tx for 3 h at room temperature. They were then incubated with the primary rabbit polyclonal anti-phospho-histone H3 (Ser10) mitosis marker (α-PH3; Upstate, Temecula, CA, USA; diluted 1:1000 in 1% NGS) overnight at room temperature with gentle shaking. After several washes with 1 mL PBS-Tx (5 × 1 min, 4 × 10 min, 6 × 1 h; room temperature), a secondary antiserum (Alexa Fluor® 488 goat anti-rabbit IgG; Invitrogen, Carlsbad, CA; diluted 1:500 in PBS-Tx) was applied overnight at room temperature in the dark with gentle shaking. The samples were washed several times with 1 mL PBS-Tx (5 × 1 min; room temperature), counterstained with the DNA-selective fluorescent dyes SYBR®Green (Invitrogen, Carlsbad, CA, USA; diluted 1:10,000 in PBS) or 4′,6-Diamidin-2-phenylindol (DAPI; Invitrogen; diluted 1:1000 in PBS) for 1 h and mounted between two cover slips in Vectashield® Mounting Medium (Vector Laboratories Inc., Burlingame, CA, USA).

For anti-caspase-3 immunolabeling, genital tracts stored in methanol were rehydrated stepwise in PBS at 4 °C (7 min each in 75, 50, 25% MeOH, 2 × PBS), washed several times with PBS-Tx (3 × 5 min) and incubated in 5% NGS for 10 min at room temperature. After replacing the blocking solution with fresh 5% NGS, the genital tracts were incubated in the blocking solution for 3 h at room temperature. Incubation with the primary antiserum (cleaved caspase-3 [Asp175] [5A1E] rabbit mAb; Cell Signaling Technology, Beverley, MA; diluted 1:50) was carried out for three nights at 4 °C with gentle shaking. After several washing steps with 1 mL PBS-Tx (5 × 5 min, 4 × 15 min, 5 × 1 h; room temperature), the secondary antiserum (Alexa Fluor® 488 goat anti-rabbit IgG; Invitrogen; diluted 1:100) was applied for three nights at 4 °C in the dark with gentle shaking. The samples were washed several times with 1 mL PBS-Tx (5 × 10 min, 3 × 1 h; room temperature), counterstained with DAPI (diluted 1:1000 in PBS) for 1 h at room temperature and mounted in Vectashield® mounting medium. The resulting signal was similar to the patterns detected with the TUNEL technique, indicating that the detected signals are specific to apoptotic cells.

### Microscopy and image processing

The samples were analyzed with the confocal laser scanning microscopes Leica TCS 5 STED (Leica Microsystems, Wetzlar, Germany) and LSM 880 (Carl Zeiss MicroImaging GmbH, Jena, Germany), and the stereomicroscope Axio Zoom V16 equipped with an Axiocam 503 color digital camera (Carl Zeiss MicroImaging GmbH). Confocal optical sections were taken at intervals ranging from 1 to 5 μm and the resulting image stacks were merged digitally into maximum projection micrographs. The colors were adjusted using Leica AS AF, v2.3.5 (Leica Microsystems), and ZEN 2012 SP1 software packages (black edition; Carl Zeiss MicroImaging GmbH). The stereomicrographs were taken at different focal planes and merged to single projections using the ZEN 2012 blue edition software version 1.1.2.0 (Carl Zeiss MicroImaging GmbH) or the Auto-Blend Layers function in Photoshop version CS 5.1 (Adobe Systems Inc., San Jose, CA, USA). All micrographs were adjusted for brightness and contrast using Photoshop CS 5.1. Final panels and diagrams were designed using Illustrator CS 5.1 (Adobe Systems Inc.).

## Additional files

Additional file 1: Apoptosis in the genital tract of the placentotrophic peripatid gen. sp. 1. Nuclei of TUNEL-positive cells are marked in orange, nuclei stained with DAPI (**A**) or SYBR®Green (**B, C**) are represented in gray. Proximal is up in all images. **A** Proximal part of the genital tract including the first five uterine chambers containing embryos of subsequent developmental stages. **B** Distal part of one uterus horn. **C** Detail of the common duct/vagina. Abbreviations: cd, common duct/vagina; ch1–ch5 and ch7, uterine chambers containing embryos of subsequent developmental stages; d, distal; p, proximal; pz, proximal proliferation zone; ut, uterus. Scale bars: A, B: 500 μm; C: 200 μm. (TIF 7280 kb)

Additional file 2: Apoptosis and cell proliferation in the genital tracts of *E. rowelli* and gen. sp. 1 and gen. sp. 2. Nuclei stained with DAPI are represented in gray, those of TUNEL-positive cells in orange, condensing DNA and chromosomes of dividing cells (α-PH3 labeling) are marked in cyan. Proximal is up in all images. **A** Overview of a genital tract of *E. rowelli*. **B** Proximal portion of the genital tracts of gen. sp. 1 (left and middle) and distal portion of the genital tract of gen. sp. 2 (right). Abbreviations: cd, common duct/vagina; ch1–ch5 and ch7, uterine chambers containing embryos of subsequent developmental stages; d, distal; dz, distal degeneration zone; oc, oocytes; ov, ovary; p, proximal; pz, proximal proliferation zone; sr, seminal receptacle; ut, uterus. Scale bars: 500 μm. (TIF 5269 kb)

Additional file 3: Apoptosis in the genital tract of *E. rowelli* detected with an antibody directed against cleaved caspase-3 (Asp175). Caspase-positive cells are illustrated in orange, cell nuclei stained with DAPI are represented in gray. Proximal is up in all images. **A** Ovary with stalked oocytes. **B** Common duct/vagina. Abbreviations: cd, common duct/vagina; oc, oocyte; ov, ovary. Scale bars: A: 500 μm; B: 200 μm. (TIF 2090 kb)

Additional file 4: TUNEL control in the proximal uterus near the implantation zone of the placentotrophic peripatid gen. sp. 1. Note that after the DNAse treatment, all cell nuclei are TUNEL-positive (orange). Abbreviations: ut, uterus. Scale bars: 200 μm. (TIF 1682 kb)

## Abbreviations

BrdU: 5-bromo-2′-deoxyuridine
cd: common duct/vagina
ch: uterine chamber
d: distal
DAPI: 4′,6-Diamidin-2-phenylindol
dz.: distal degeneration zone
EdU: 5-ethynyl-2′-deoxyuridine
em: fully developed embryo
ge: germinal epithelium
gen. sp.: undescribed species
iz: implantation zone
mg/ml: milligram/milliliter
mo: morula
mol/L: mole per liter
NGS: Normal goat serum
oc: oocyte
of: ovarian funnel
ov: ovary
p: proximal
PBS: phosphate-buffered saline
PBS-Tx: phosphate-buffered saline containing Triton X-100
PH3: phospho-histone H3
pz: proximal proliferation zone
sr: seminal receptacle
TUNEL: terminal deoxinucleotidyl transferase-mediated dUTP nick end labeling
ut: uterus
μL: microliter
