# Supplementary figures and images for: Evidence for cell turnover as the mechanism responsible for the transport of embryos towards the vagina in viviparous onychophorans (velvet worms)
Source: Front Zool. 2019 Jun 7;16:16. doi: 10.1186/s12983-019-0317-x (PMC6555992; doi:10.1186/s12983-019-0317-x)

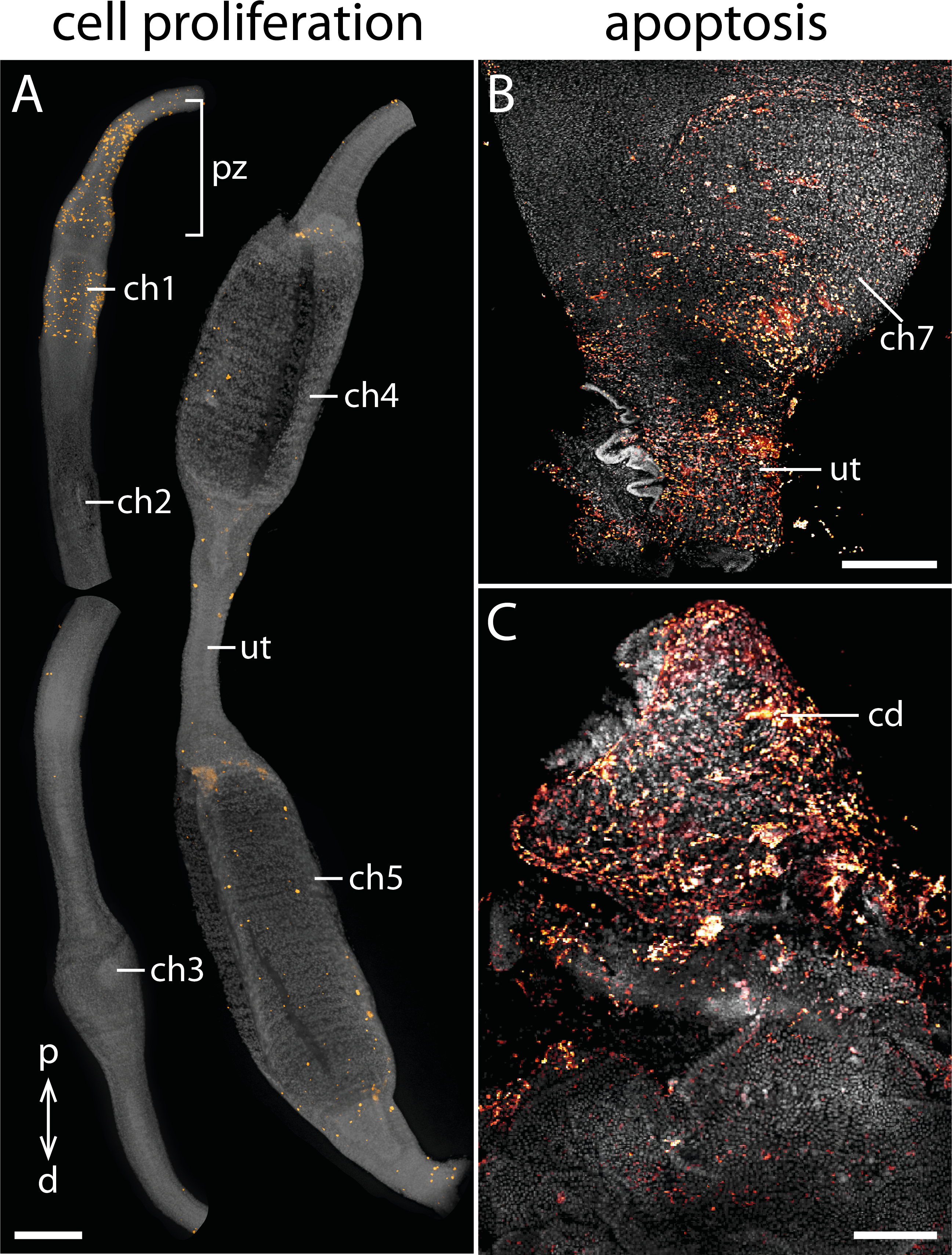

Supplement: Supplementary file 1 — Apoptosis in the genital tract of the placentotrophic peripatid gen. sp. 1. Nuclei of TUNEL-positive cells are marked in orange, nuclei stained with DAPI (A) or SYBR®Green (B, C) are represented in gray. Proximal is up in all images. A Proximal part of the genital tract including the first five uterine chambers containing embryos of subsequent developmental stages. B Distal part of one uterus horn. C Detail of the common duct/vagina. Abbreviations: cd, common duct/vagina; ch1–ch5 and ch7, uterine chambers containing embryos of subsequent developmental stages; d, distal; p, proximal; pz, proximal proliferation zone; ut, uterus. Scale bars: A, B: 500 μm; C: 200 μm. (TIF 7280 kb) [file 12983_2019_317_MOESM1_ESM.tif]

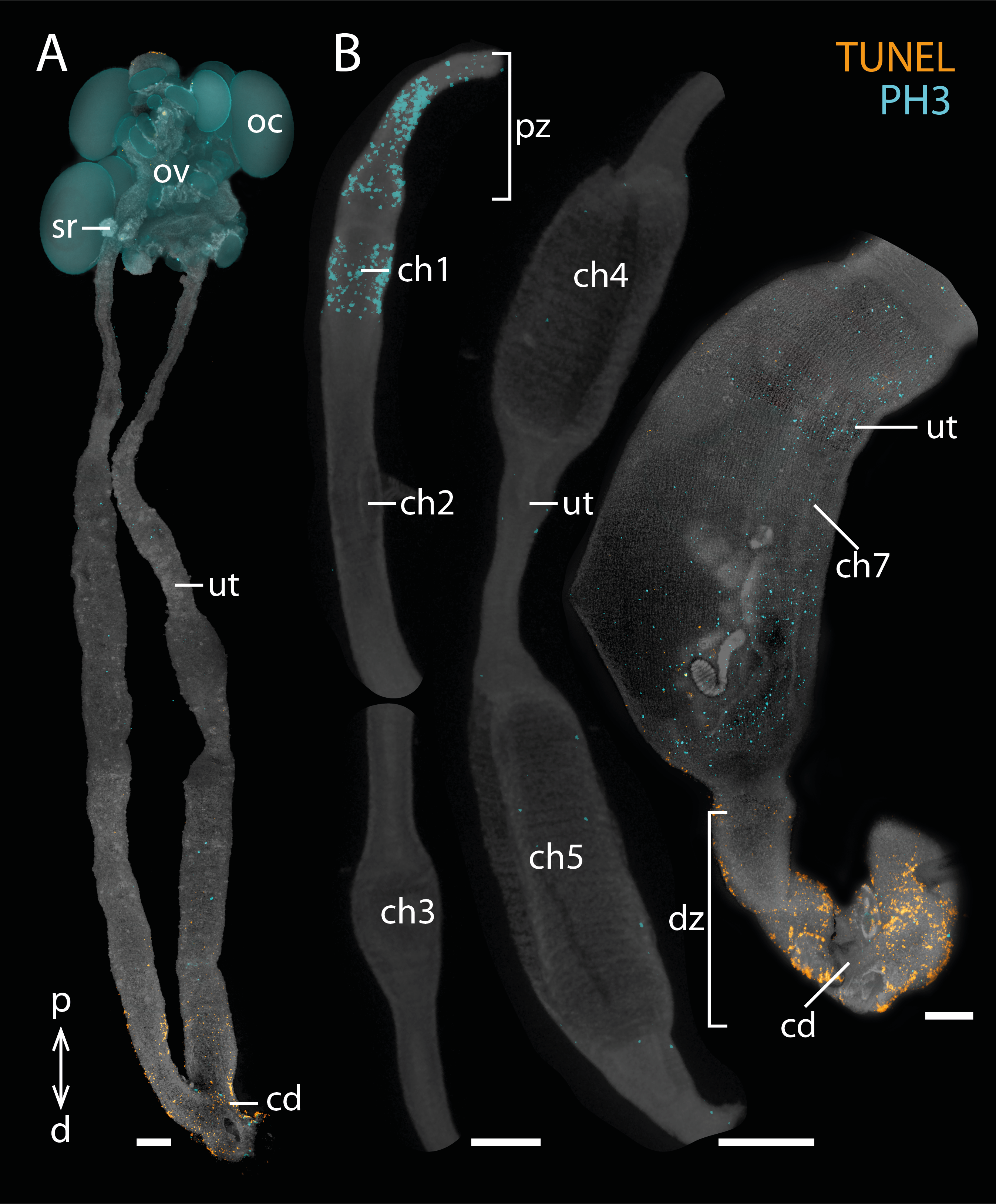

Supplement: Supplementary file 2 — Apoptosis and cell proliferation in the genital tracts of E. rowelli and gen. sp. 1 and gen. sp. 2. Nuclei stained with DAPI are represented in gray, those of TUNEL-positive cells in orange, condensing DNA and chromosomes of dividing cells (α-PH3 labeling) are marked in cyan. Proximal is up in all images. A Overview of a genital tract of E. rowelli. B Proximal portion of the genital tracts of gen. sp. 1 (left and middle) and distal portion of the genital tract of gen. sp. 2 (right). Abbreviations: cd, common duct/vagina; ch1–ch5 and ch7, uterine chambers containing embryos of subsequent developmental stages; d, distal; dz, distal degeneration zone; oc, oocytes; ov, ovary; p, proximal; pz, proximal proliferation zone; sr, seminal receptacle; ut, uterus. Scale bars: 500 μm. (TIF 5269 kb) [file 12983_2019_317_MOESM2_ESM.tif]

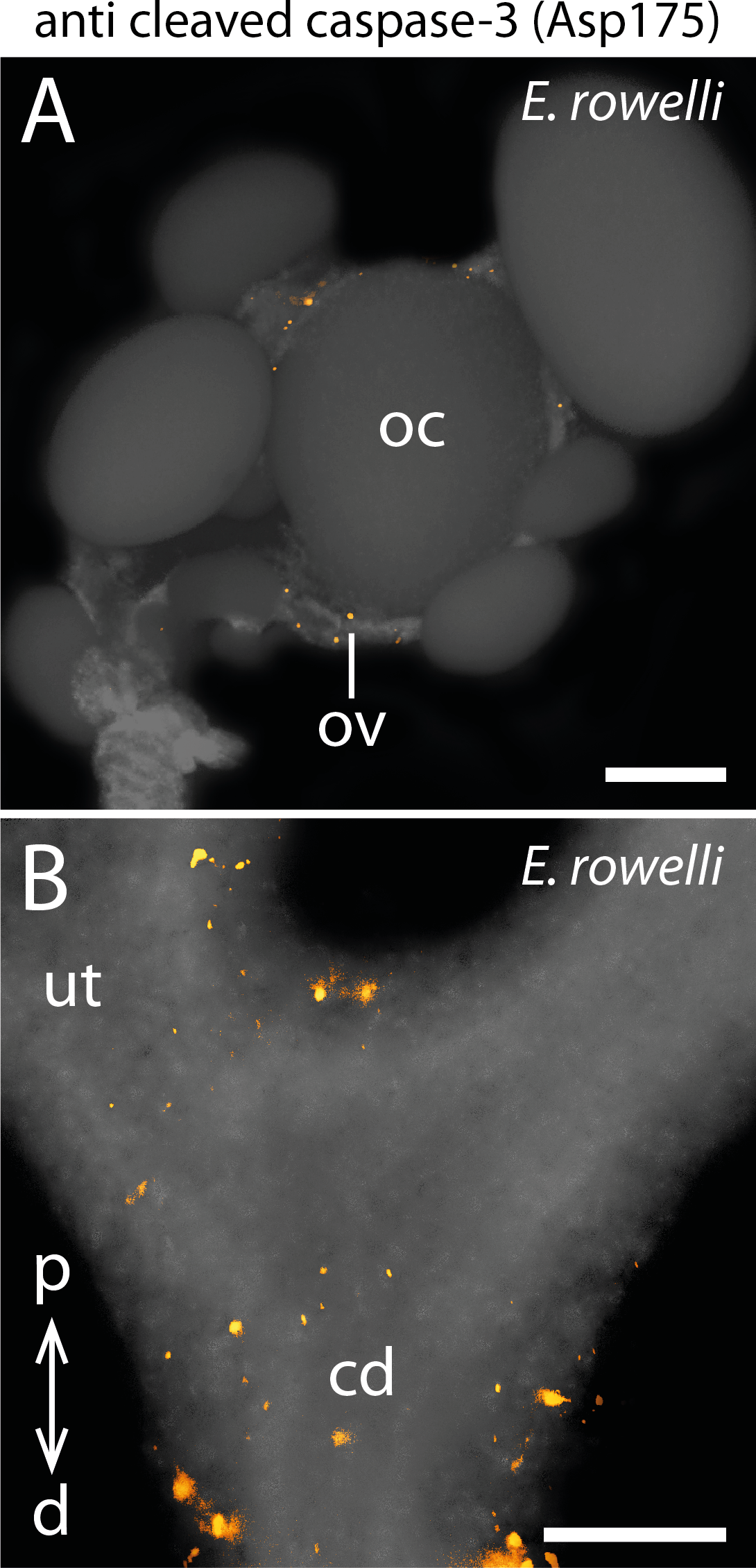

Supplement: Supplementary file 3 — Apoptosis in the genital tract of E. rowelli detected with an antibody directed against cleaved caspase-3 (Asp175). Caspase-positive cells are illustrated in orange, cell nuclei stained with DAPI are represented in gray. Proximal is up in all images. A Ovary with stalked oocytes. B Common duct/vagina. Abbreviations: cd, common duct/vagina; oc, oocyte; ov, ovary. Scale bars: A: 500 μm; B: 200 μm. (TIF 2090 kb) [file 12983_2019_317_MOESM3_ESM.tif]

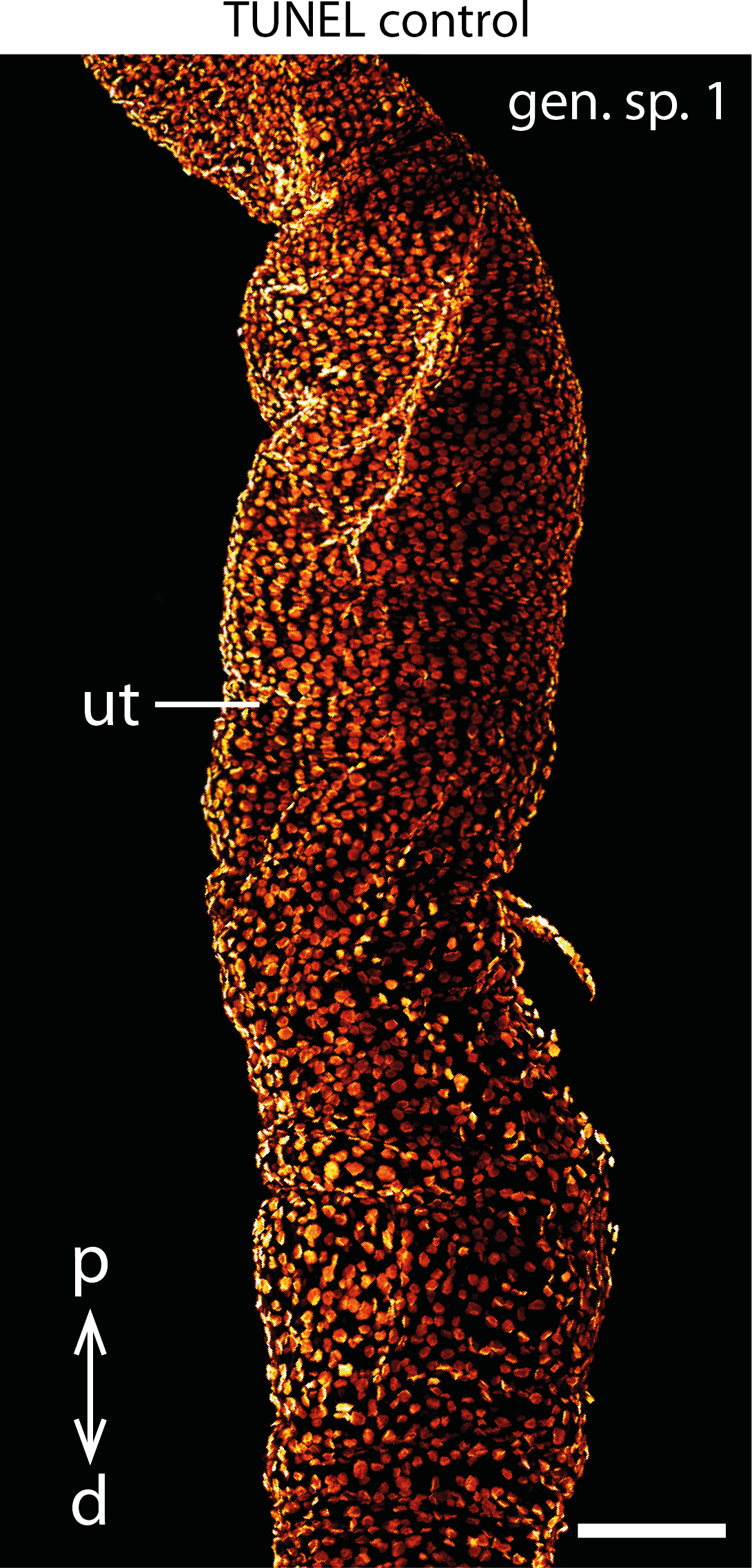

Supplement: Supplementary file 4 — TUNEL control in the proximal uterus near the implantation zone of the placentotrophic peripatid gen. sp. 1. Note that after the DNAse treatment, all cell nuclei are TUNEL-positive (orange). Abbreviations: ut, uterus. Scale bars: 200 μm. (TIF 1682 kb) [file 12983_2019_317_MOESM4_ESM.tif]
